# 

**Published:** 1784

**Authors:** 


### PROMOTIONS.

Lately, Mr. George Forster, F. R. S. to be professor of natural history at Wilna, in Poland.

1783.

1783. *Feb.* 11. Charles Peter Martin de Sarguemines, M. D. to be fellow of the Royal College of Phyficians at Nancy.

1784. *May* 31. Mr. Nicholas Archer to be furgeon of the fixth regiment of foot.

*June.* Dr. Frederick Michaelis to be phyfician to the Landgrave, and profeffor of the practice of phyfic at Heffe Caffel.—25. Mr. Samuel Pierfon, to be furgeon of the 26th regiment of foot.

*July.* Francis Riollay, of Hertford College, Oxford, M. B. to be M. D.—22. Mr. J. Earle to be furgeon, and Mr. Ludlow Harvey to be affiftant furgeon to St. Bartholomew's Hofpital.

*Auguft.*—Mr. William Rae to be dentift to His Royal Highnefs the Prince of Wales.—3. Dr. Robert Grant of Invernefs, to be fellow of the Royal College of Phyficians at Edinburgh.—9. Dr. David Pitcairne, and Dr. Francis Riollay, to be candidates, and Dr. Thomas Watfon, Dr. William Woodville, and Dr. John Relph, to be licentiates of the Royal College of Phyficians of London.—26. Dr. J. G. Caulet to be phyfician to St. Bartholomew's Hofpital.—Mr. George Mitchell to be furgeon of the Eaftern Difpenfary, in White Chapel.

*Sept.* 11. Mr. Mac Intyre, to be Surgeon of the Garrifon at Annapolis Royal. — Mr. Thomas Irwin, late Surgeon of the 39th regiment of foot, to be Surgeon to the Garrifon at Halifax. — Dr. John Calef, to be Surgeon to the Garrifon at New Brunfwick. — Dr. William Smith, to be Surgeon to the Garrifon at Cape Breton. — Mr. Thomas Walker, to be Surgeon to the Garrifon at St. John's Ifland.

---

### DEATHS.

Lately, at Montpellier, Peter Cuffon, M. D. a learned Phyfician and Botanift, and member of the Royal Society of Sciences of that city. He enjoyed the friendfhip of the late M. de Sauvages, and had the honour of affifting him in his *Nofologia Methodica.* The fixth order of the firft clafs of difeafes in that celebrated work was written entirely by M. Cuffon. When he was a young man, he travelled to Majorca and Spain, and brought back with him an excellent collection of the plants of thofe countries and the Pyrenées. To this anecdote of himfelf, which he formerly related to the editor of this Journal, he added, that foon after his return from from his travels, an old female relation, who lived with him, took an opportunity one day, when he was from home, to clean and rummage his ſtudy. In the courſe of this buſineſs ſhe turned over his dried plants, and conſidering them as ſo much uſeleſs lumber, threw the whole of his fine collection into the ſtreet. For ſeveral years before his death he was employed in a great work on the umbelliferous plants, which he is ſaid to have left in a ſtate fit for the preſs. Dr. Brouſſonet, of Paris, has undertaken to write an account of his life. — At Leyden, Dr. John David Hahn, and Dr. Walter Van Doeveren, two eminent Medical Profeſſors, of whom we hope to procure ſome authentic anecdotes. — At Canton in China, Mr. James Dewar, Surgeon of the Engliſh factory at that place.

1782. At Dijon, M. Fournier, M. D. author of a work on hectic fevers, of which we formerly gave ſome account (vol. II. p. 280.)

*Auguſt* 17. At Berlin, Andrew Sigiſmund Marggraf, a celebrated chemical writer, and Director of the Phyſical Claſs of the Academy of Sciences at Berlin.

1783. *Jan.* 7. John Targioni Tozzetti, M. D. Profeſſor of Botany at Florence, and author of of a great number of learned works in phyfic and natural hiftory. He was born at Florence Sept. 11, 1712.

1784. *Jan.* 28. At Wirtemberg, John Erneft Zeicher, M. D. Profeffor of Mathematics, and author of feveral mathematical and botanical differtations. He was born at Weiffenfels in 1720.

*Feb.* 3. At Leipfic, in his 31ft year, Chriftian Frederick Ludwig, M. D. fon of the late celebrated Profeffor of that name. In 1781, after having vifited Paris, he paffed a few months in this country, and foon after his return to Leipfic was appointed to a Medical Profefforfhip. In the quarterly catalogue of our prefent number will be found the title of a differtation he publifhed on that occafion.—27. Anthony William Plaz, M. D. Profeffor of Botany in the Univerfity of Leipfic. He was born in that city January 1, 1708, and was author of feveral botanic differtations.

*June.* In Warwick Court, Holborn, Mr. John Wynde, Apothecary. —21. Aged 58 years, Cheney Hart, M. D. in the commiffion of the peace for the county of Salop, and fenior Phyfician of the Infirmary at Shrewfbury. He was author of an inaugural differtation *de Cor-*

*tice*

*tice Peruviano*, printed at Edinburgh in 1748, and of an account of the effects of electricity in the County Hospital at Shrewsbury, published in the 48 and 49th vols. of the Philosophical Transactions. — 29. In Red-Lion Square, Holbourn, Mr. Stafford Crane, one of the Court of Examiners of the Corporation of Surgeons, and Surgeon to St. Bartholomew's Hospital.

*July* 5. At Dover in Kent, Mr. John Broadley, surgeon. — 9. At Medewi in Sweden, aged 50 years, Sir Thorbern Bergman, Knight of the order of Wasa, Professor of Chemistry at Upsal, Fellow of the Royal Society of London, and of almost all the learned societies in Europe. We shall probably soon have it in our power to give some account of this celebrated philosopher, to whose genius and labours the science of chemistry is singularly indebted. — 10. At Olney in Buckinghamshire, Mr. Grindon, Apothecary. — At Oxford, aged 85 years, Mr. Anthony Rawlins, formerly an apothecary in that city. — 25. At Bath, Matthew Dobson, M. D. F. R. S. author of an inaugural dissertation *de Menstruis*, printed at Edinburgh in 1756; of a commentary on fixed air, printed at Chester in 1759; and of several valuable papers in the Philosophical Transactions, Medical

Obſervations and Inquiries, and other collec-tions. About three years before his death, he removed from Liverpool (where he had long been in conſiderable practice) to Bath. His wife, who ſurvives him, has acquired great reputation in the literary world by a very ele-gant life of Petrarch, and other works. We ſhall be thankful to any perſon who will enable us to give a farther account of this ingenious and reſpectable phyſician.

*Auguſt.* At Paris, John Francis Clement Morand, (ſon of the late celebrated Surgeon of that name) Doctor Regent of the Faculty of Phyſic, and member of the Royal Academy of Sciences of that city; Fellow of the Royal Society of London, and of ſeveral other learn-ed inſtitutions. He was born at Paris April 28, 1726; and in 1752 acquired much reputation by his account of a remarkable inſtance of *Mollities oſſium* in a woman named Supiot. He was alſo author of a letter, publiſhed in 1755, on the *Vectis* of Roonhuyſen, and of ſeveral eſſays in the Memoirs of the Academy of Sci-ences, and *Journal de Medecine.* — At Paris, Anthony Deſtremeau, member of the Academy of Surgery, and Accoucheur to her R. H. the Counteſs d'Artois. — At Rouen, John Peter

 David,

David, a Surgeon of great ability and eminence. He was a native of the *Pays de Gex*, and married the only daughter of the late M. le Cat, whom he ſucceeded as Profeſſor of Anatomy and Surgery, and principal Surgeon of the Hotel Dieu at Rouen. He was author of an ingenious diſſertation on the mechaniſm of reſpiration, printed at Paris in 1766; of an eſſay *de lactis origine*, &c. publiſhed in the 7th vol. of the Haerlem Memoirs; of obſervations on a diſeaſe of the bones, known by the name of *Necroſis*, of which we formerly gave an account (vol. III. p. 369), and of ſome other works, publiſhed either ſeparately or in the Memoirs of the Academy of Surgery at Paris, of which he became a member in 1764. — At Paris, Laurence Garnier, M. D. ſenior member of the College of Phyſicians at Lyons. — 9. In Queen Square, London, aged 50 years, Richard Tyſon, M. D. in the Univerſity of Cambridge, Fellow of the Royal College of Phyſicians, and Phyſician to St. Bartholomew's Hoſpital. — 13. At Weſtmead, near Llangharne in Carmarthenſhire, John Morgan, M. D. author of an inaugural diſſertation *de dolore capitis*, printed at Edinburgh in 1769. — 27. At Saliſbury, aged 29 years, Mr. Thomas

 Fawler,

Fawler, apothecary at Clapham in Surry.—30. At Binfield, in Berkſhire, aged 38 years, Michael Teighe, M. D. in the Univerſity of Rheims, F. R. S. member of the Royal College of Phyſicians of London, and formerly phyſician to the Weſtminſter General Diſpenſary.—In Old-ſtreet, London, Mr. Thomas Godman, ſurgeon to the Charter-houſe; author of "A Remonſtrance againſt the miſchievous abuſe of Phlebotomy," publiſhed in 1747.

*Sept.* 2. At Bridlington, in Yorkſhire, aged 72 years, Mr. Thomas Boardman, ſurgeon and apothecary.—7. Mr. John Dray, ſurgeon and apothecary, at Dover, in Kent.

---

### SECTION IV.

### QUARTERLY CATALOGUE.

1. AN eſſay on the management and nurſing of children in the early period of infancy; and on the treatment and rule of conduct requiſite for the mother during pregnancy and in lying-in: including the diſeaſes to which the mother and child are liable; with the method of curing, and particularly of preventing many many of thoſe diſeaſes. The whole addreſſed, as well to the Medical Faculty, as to the public at large; and purpoſely adapted to a female comprehenſion, in a manner perfectly conſiſtent with the delicacy of the ſex. By *William Moſs*, ſurgeon, 8vo. *Johnſon*, London, 1781. 372 pages. 5s.

This volume is the production of a judicious and intelligent writer, and though deſigned for the public at large, contains a variety of obſervations which entitle it to the attention of medical practitioners.

2. Curſory Remarks on the nature and cauſe of the Marine Scurvy, ſhewing that that diſtemper may not only be prevented, but probably cured on board ſhips at any diſtance from land. 4to. *Baldwin*, London, 1783. 83 pages.

This work, we are told, was written ſo long ago as the year 1771, and was then put into the hands of Dr. Huck Saunders. The author of it (Mr. Sherwin, ſurgeon at Endfield) was formerly ſurgeon of an Eaſt-Indiaman. He recommends the meal of malted wheat in preference to ſweet wort, and his obſervations on this and other articles of the diet of ſeamen, are extremely judicious.

3. An Account of the Life and Writings of the celebrated Dr. Archibald Pitcairne, delivered as the Harveian Oration at Edinburgh, for the year 1781. By *Charles Webſter*, M. D. phyſician to the Public Diſpenſary, of the Royal College of Phyſicians, Edinburgh, of the Royal Society of Medicine, Paris, &c. 8vo. Edinburgh. 42 pages.

This elegant tribute to the memory of Dr. Archibald Pitcairne, is inſcribed to his learned kinſman, the preſent worthy Preſident of the College of Phyſicians of London. The moſt intereſting particulars of his life are recorded, and in reviewing his writings, Dr. Webſter takes occaſion to trace the outlines of the doctrines which characterized the mechanical phyſicians of the laſt century.

4. An Eſſay on the bite of a mad dog, in which the claim to infallibility of the principal preſervative remedies againſt the Hydrophobia is examined. By *John Berkenhout*, M. D. 8vo. *Baldwin*, London, 1783. 1s. 6d.

The chief deſign of this work, which contains many judicious obſervations, ſeems to be to induce the public to withdraw their confidence from the remedies hitherto recommended as preſervatives againſt the Hydrophobia.

5. An

5. An improved method of opening the Temporal Artery; alſo a new propoſal for extracting the Cataract; with deſcriptions and delineations of the inſtruments contrived for both operations, by the author * when a ſtudent at Edinburgh. To which are now added, a miſcellaneous introduction, and caſes and obſervations, chiefly tending to illuſtrate the good effects of arteriotomy in various diſeaſes of the head. By the ſame author. 8vo. *Robſon*, London, 1783. 213 pages.

6. A familiar Medical Survey of Liverpool, addreſſed to the inhabitants at large; containing obſervations on the ſituation of the town, the qualities and influence of the air, the employments and manner of living of the inhabitants, the water, and other natural and occaſional circumſtances, whereby the health of the inhabitants is liable to be particularly affected. With an account of the diſeaſes moſt peculiar to the town, and the rules to be obſerved for their prevention and cure: including obſervations on the cure of conſumptions. The whole rendered perfectly plain and familiar. By *W. Moſs*, ſurgeon at Liverpool. 8vo. Liverpool, 1784. 2s,

* William Butter, M. D.

7. The Caſe of the Rev. Dr. Harwood; an obſtinate Palſy of above two years duration, greatly relieved by Electricity. By *Edward Harwood, D. D.* 8vo. *Buckland*, London, 1784. 42 pages. 1s.

8. Lectures on the Gravid Uterus, and Midwifery; as taught and practiced by the late Dr. *Hunter*: (with the Medical terms in Midwifery explained for the benefit of female practitioners); by one who ſtudied under him. 8vo. *Flexney*, London, 1783. 64 pages. 1s. 6d.

9. Some new hints, relative to the recovery of perſons drowned, and apparently dead; with a view to render that practice more generally ſucceſsful. By *John Fuller*, ſurgeon at Ayton, Berwickſhire. 8vo. *Cadell*, London, 1784. 32 pages. 1s.

10. Diſſertatio Medica Inauguralis de Veſicantium uſu in variis morbis tractandis. Auctore *Henrico Donly*, Anglo; Societat. Phyſic. Chirurg. Edinb. Socio. 4to. Lugd. Batav. 1784. p. 34.

11. Diſſertatio Medica Inauguralis de Lactis Secretione in Puerperis. Auctore *Joanne Aikin*, Anglo; Soc. Reg. Med. Edin. Soc. Phyſ. Literar. Mancun. ſocio honorario. 4to. Lugd. Batav. 1784. p. 28.

12. De Suffufionis per acum Curatione. Auctore *Chriftiano Frederico Ludwig.* 4to. Lipfiæ, 1783. p. 23.

13. Grifte-Tal ofver Valborne-Herrn Herr Carl von Linnè, M. D. &c. *i. e.* a Funeral Oration in honour of the illuftrious Charles von Linné, M. D. profeffor of Phyfic and Botany in the univerfity of Upfal, &c. delivered in the cathedral of Upfal, November 30, 1783, when the ceremony of breaking the coat of arms of the family of Linné, on account of the male line of that family being extinct, was performed by *David Schulz von Schulzenheim.* 8vo. Upfala, 1784. 42 pages.

To the account we formerly gave of the younger Linnæus (vol. IV. p. 433) we are now able to add, from the work before us, that he was born at Fahlun, in Dalecarlia, January 20, 1741; and was appointed demonftrator of Botany at Upfal, in 1759, and profeffor in 1763. His biographer mentions his travels into France and England, and gives a fummary account of his writings. An engraving of the coat of arms of the family of Linné is prefixed to the work.

14. Eloge de Jean Palfyn, Chirurgien et Profeffeur en Chirurgie de la ville de Gand, &c. *i. e.* Eulogium of John Palfyn, furgeon and and professor of Surgery of the city of Ghent, pronounced by M. Van Dueren, licentiate in Physic, on the 11th of February, 1783, at the opening of the mausoleum erected * to his memory in the parish church of St. James, by the College of Physicians at Ghent. 4to. Ghent, 1783. 14 pages.

15. Memoire sur l'inoculation de la Peste, avec la description de trois poudres fumigatoires. *i. e.* Essay on the inoculation of the Plague, with a description of three fumigating powders. By *D. Samoilowitz*, M. D. &c. 8vo. Paris, 1782.

We have already had occasion (vol. III. p. 329) to take notice of a work of this author's on the external use of ice in the Plague. In the present essay he contends, that no person can have the Plague twice, and asserts that by inoculation the disease is disarmed of its malignancy.

* With the following inscription:

D. O. M.

Et piis manibus

Joannis Palfyn

Scriptis anatomicis et chirurgicis per Europam clari

Obiit die 7 Februarii, 1733. Ætatis suæ 78.

Posuit Collegium Medicum Gandavense

1783.

16. Lettre á l'Academie de Dijon, avec reponse a ce qui a parû douteux dans le memoire sur l'inoculation de la Peste. *i. e.* Letter to the academy of Dijon, with an answer to what appeared doubtful in the essay on the inoculation of the Plague. By *D. Samoilowitz*, M. D. &c. 8vo. Paris, 1783. 77 pages.

This letter is intended to obviate some objections which have been offered to the author's proposal for inoculating the Plague. He adduces several arguments to prove that the Plague, even in Egypt, is perfectly independent of any particular state of the atmosphere; that it is not influenced by climate or season, as it has been known to rage with equal fury in the coldest winter and the hottest summer; and that it is propagated only by the immediate contact of solid bodies. He contends, that its effects are confined to the human species, and in proof of this relates, that at Kiow, during the prevalence of the Plague, a cat belonging to a house, in which every body had died of the Plague, went into another house and conveyed the infection to the family there, but was not itself affected by the disease.

17. *Joh. Alex. Brambilla* August. Imp. Joseph. II. Chir. ord. Instrumentarium Chirurgicum Viennenſe. Folio. Vienna, 1782, with 65 plates.

The Emperor having inſtituted a ſchool of ſurgery at Gumpendorff, near Vienna, ordered all the inſtruments of ſurgery that are now uſed to be procured for it from Paris and London. This collection is the ſubject of the preſent work.

18. Precis Hiſtorique des faits relatifs au Magnetiſme Animal, juſqu'en Avril 1781. *i. e.* Hiſtorical account of the facts relative to animal magnetiſm, to April 1781. By M. *Meſmer*, M. D. of the faculty of phyſic at Vienna. 8vo. Paris, 1781. 228 pages.

19. Obſervation tres importante ſur les effects du Magnetiſme Animal, *i. e.* A very important obſervation on the effects of animal magnetiſm. By M. *de Bourzeis*, M. D. phyſician in ordinary to the king. 8vo. Paris, 1783, 26 pages.

This writer complains of Mr. Meſmer's having unfairly gotten one of his patients from him. The patient died.

20. Lettre ſur la decouverte du Magnetiſme Animal. *i. e.* A letter on the diſcovery of animal magnetiſm to M. Court de Gibelin, Cenſor Royal, &c. By the Reverend Father *Hervier*,

*Hervier*, Doctor of the Sorbonne. 8vo. Paris, 1784. 48 pages.

21. Mesmer blessé, ou reponse a la lettre du Reverend Pere Hervier sur le Magnetisme Animal. *i. e.* Mesmer wounded, or an answer to the letter of the Reverend Father Hervier on animal magnetism. By M. —— 8vo. Paris, 1784. p. 34.

22. Mesmer justifié. *i. e.* Mesmer justified. 8vo. Paris. 1784. p. 46.

This pretended justification is ironical, and is written to excite a laugh at the expence of Mesmer.

23. Lettre de l'auteur du Monde primitif a Messieurs ses Souscripteurs sur le Magnetisme Animal. *i. e.* Letter from the author of the Primitive World to his subscribers concerning animal magnetism. 4to. Paris, 1784. 48 pages.

The learned M. Court de Gibelin here relates his own case, and attributes his recovery to M. Mesmer. The testimony of so respectable a writer would probably have been very useful to Mesmer, but unfortunately M. de Gibelin died in May 1784, very soon after the publication of his pamphlet.

24. Me-

24. Memoire dans lequel on demontre les phenomenes du Mesmerisme ; nouvelle edition, precedée d'une lettre sur le secret de M. Mesmer, avec figures. *i. e.* Memoir in which are demonstrated the phenomena of Mesmerism. A new edition, preceded by a letter concerning the secret of M. Mesmer, with figures. By M. *Retz*, quarterly physician in ordinary to the king, and formerly physician to the marine hospitals. 8vo. Paris, 1784.

25. Rapport des commissaires de la Societé Royale de Medecine, nommés par le Roi pour faire l'examen du Magnetisme Animal. *i. e.* Report of the Committee of the Royal Medical Society appointed by the King to inquire into animal magnetism. 4to. Paris, 1784. 39 pages.

26. Detail des cures operées a Buzancy, prés Soissons, par le Magnetisme Animal. *i. e.* Account of the cures performed at Buzancy, near Soissons, by animal magnetism. 12mo. Soissons, 1784. 44 pages.

27. Recherches et doutes sur le Magnetisme Animal. *i. e.* Researches and doubts concerning animal magnetism. By M. *Thouret*, doctor regent of the faculty of physic, and member of the the Royal Medical Society at Paris. 8vo. Paris, 1784. 251 pages.

28. Richtiger Gebrauch des bley extracts in æuſerlichen Schædens, &c. *i. e.* Of the proper uſe of extract of lead in external diſeaſes. 8vo. Halle, 1783.

29. Kurze Nachricht von der epidemiſchen ſchnupfen krankheit und der Beſchaffenheit der Luft 1781 und 1782. *i. e.* A ſhort account of the epidemical catarrhal fever, and of the ſtate of the air in the years 1781 and 1782. By a phyſician at Hamburg. 8vo. Hamburg, 1782.

30. Beſcreibung der Epidemien, welche im Frujahr des 1782 jahres in meheren gegenden von Europa geherrſchet und unter dem namen der Ruſſiſchen krankheit bekannt geworden. *i. e.* Deſcription of the epidemic which in the ſpring of the year 1782 prevailed in many countries of Europe, and was known under the name of the Ruſſian diſeaſe. 8vo. Leipſic, 1782.

31. Diſſertatio hiſtoriam Catarrhi Epidemici A. 1782 ſiſtens, Preſide Laurent. Crell, reſp. et auct. *Jo. Fr. Languth*, 8vo. Helmſtadt, 1782. p. 164.

32. Fundamenta Chemiæ theoretico-practicæ

poſita posita a D. *J. W. Baumer*. 8vo. Giessæ 1783. p. 528.

33. Metodo per curare sicuramente l'idropisia, &c. *i. e.* Method of curing with safety the dropsy; with observations on the use of the Peruvian bark, on the bite of the viper, and on the cure of madness. By *John Baptist Moreali*, M. D. 8vo. Venice, 1784.

34. Atmospheræ pressio varia, observationibus baroscopicis propriis et alienis, quæsita a *Cælestino Steiglehner*, S.S. Theolog. & Phil. Doct. S. El. Bavaro-Palatini Cons. Eccles. Physices Theor. et Experim. ac Meteorol. Prof. quam D. *Henr. Maria Leveling*, tentamen inaugurale publicum subiret. 4to. Ingolstadt 1783. p. 58.

35. Examen physico-chemicum Acidularum Freudenthalensium in Silesia, quod publicæ disquisitioni submittit *Sebast. Durer*. 8vo. Vienna, 1782.

36. Chirurgische Wahrnehmungen. *i. e.* Chirurgical Observations. Part II. by *Adolphus Frederick Vogel*, M. D. 8vo. Lubeck, 1780. 88 pages.

37. Pharmacopeia Manualis reformata, edita a *Benedicto Majon*, Hispano, in Universitate Genuensi Chemiæ demonstratore. 8vo. Genoa, 1784.

